# Treatment of an Esophageal Stricture in a 1-Month-Old Miniature Shetland Colt

**DOI:** 10.1155/2017/3069419

**Published:** 2017-11-20

**Authors:** P. Nijdam, C. Elmas, M. C. Fugazzola

**Affiliations:** Burg Müggenhausen Equine Clinic GmbH, Heimerzheimer Straße 18, 53919 Weilerswist, Germany

## Abstract

A 1-month-old Miniature Shetland colt was presented to the Burg Müggenhausen Equine Clinic. Primary complaints were regurgitation of milk, bilateral white nasal discharge, and weakness. Physical examination, endoscopy of the esophagus, and contrast radiography led to the diagnosis of an esophageal stricture and aspiration pneumonia. Surgical treatment by means of an esophagomyotomy was performed. The foal improved only temporarily and multiple sessions of endoscopic balloon dilation of the esophagus were performed afterwards. 12 months after the final treatment the foal was healthy and had no complaints regarding esophageal function.

## 1. Introduction

Esophageal stricture is a narrowing of the lumen of the esophagus [1]. Strictures usually form as a result of external or internal trauma to the esophageal wall [2]. Congenital esophageal strictures, though rare, have also been documented [3, 4]. Three types of esophageal strictures are recognized: type 1: lesions involving the adventitia and tunica muscularis; type 2: esophageal rings or webs that involve mucosa and submucosa; type 3: annular stenosis that involves all layers of the esophageal wall [2].

Esophageal strictures usually lead to impaired passage of food. Nasal discharge, salivation, coughing, discomfort and extension of the head and neck may all occur [5]. Aspiration pneumonia, pleuritis, esophageal ulceration, and formation of diverticula are common complications [6].

Several imaging modalities may aid in the diagnosis. Endoscopy, contrast radiography, fluoroscopy, and ultrasonography can be used to examine the esophagus [4, 7].

Various treatment options are available to prevent stricture formation [1, 2, 8]. After esophageal wall damage, the greatest reduction in lumen diameter occurs in the first 15 days [5]. Preventive treatment should therefore be aimed at the initial 15 days and consist of anti-inflammatory medication and feeding soft feeds only [9].

Alternative treatment methods should be applied for existing strictures and include bougienage, balloon dilation, and surgical intervention. Bougienage refers to dilation the esophagus by passing a rigid instrument through it. The bougie mainly delivers shearing forces, which may easily lead to damage to the esophageal wall [4]. More recently the use of balloon dilation has also been described, in which the stricture is dilated by inflating a balloon [10–12]. Balloon dilation delivers mainly radial forces upon the esophageal wall and therefore the risk of damaging the esophagus is smaller.

Esophageal strictures can also be treated surgically. Commonly used techniques include esophagomyotomy for type 1 strictures and partial resection, patch grafting, and mucosal fenestration for type 2 and 3 strictures [1, 13]. Esophageal surgery is technically demanding and often associated with complications, such as dehiscence, reformation of strictures, or infection.

Overall, prognosis for esophageal strictures is guarded and depends on the type and degree of stricture formation [1, 5, 6].

## 2. Case Presentation

A 1-month-old Miniature Shetland colt weighing 14 kilograms was presented to the Burg Müggenhausen Equine Clinic for emergency treatment. The foal had been regurgitating milk after nursing, had white bilateral nasal discharge, and had been weak since the morning of the day of admission.

Physical examination showed a respiratory rate of 28/minute, heart rate of 80/minute, and a rectal temperature of 38,0°C. Lung auscultation revealed bilateral crackles and wheezes. A complete blood count (CBC) and plasma biochemical profile showed the following abnormalities (Table 1).

At endoscopy white fluid was visible in the pharynx and esophagus. Both entrances to the guttural pouches were draining white fluid. Presumably the white fluid was milk. The endoscope could be passed 4-5 cm into the esophagus.

**Figure 1:**
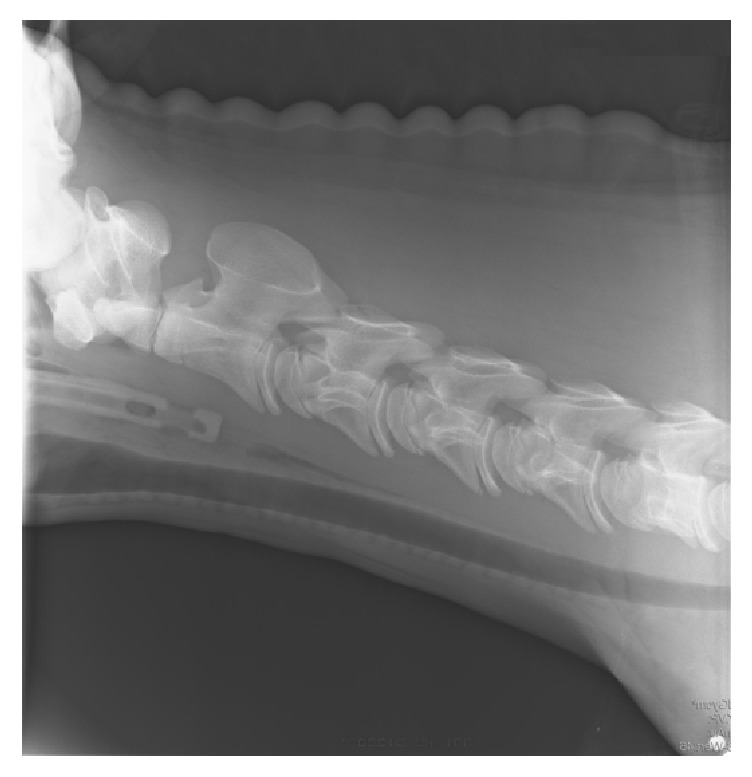
Lateral radiographic view of the cervical area. The nasogastric tube can be seen advanced up to the stricture site at approximately the second cervical vertebra.

**Figure 2:**
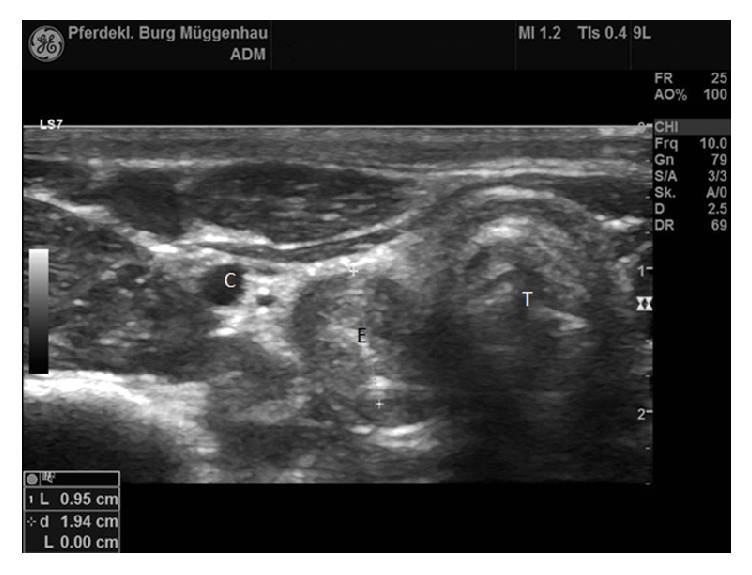
Ultrasound image of the esophagus cranial to the esophageal stricture. Esophageal diameter is 9,5 mm. Left in the image is dorsal. C: carotid artery. E: esophagus. T: trachea (probe frequency 10 MHz, depth 2,5 cm).

**Figure 3:**
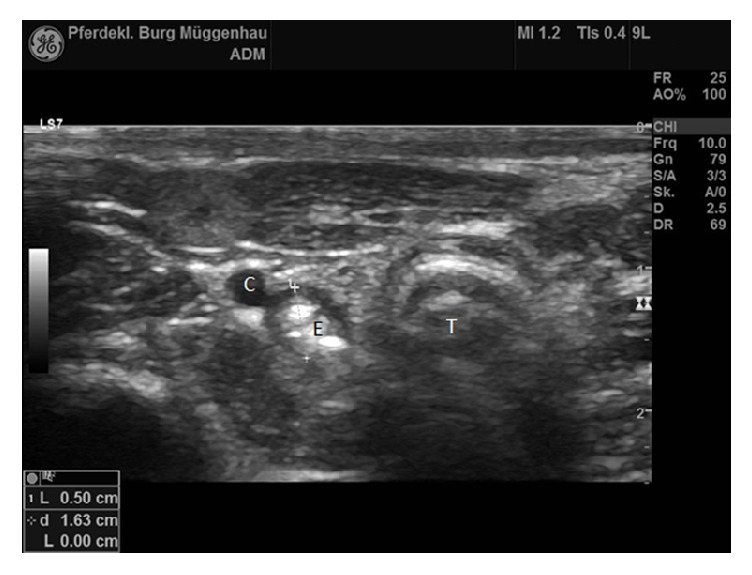
Ultrasound image of the stricture site. Left in the image is dorsal. A substantial decrease in diameter compared to being immediately cranial to the stricture can be observed. C: carotid artery. E: esophagus. T: trachea (probe frequency 10 MHz, depth 2,5 cm).

Contrast radiography of the esophagus was performed. A nasogastric tube was passed into the esophagus and subsequently 50 ml of contrast medium (Micropaque®, Guerbert GmbH) was inserted. Contrast medium and the nasogastric tube itself could be seen up to approximately 4 cm caudal to the larynx, where a narrowing of the esophageal lumen was visible at the second cervical vertebra (Figure 1). Thoracic radiographs showed a prominent bronchial pattern. Ultrasonography of the cervical part of the esophagus revealed a distinct narrowing of the diameter from 9,5 mm immediately cranial to the stricture to 5 mm at the stricture itself. Involvement of only the adventitia and tunica muscularis was suspected (Figures 2 and 3).

Based on imaging, a type 1 esophageal stricture was diagnosed. The foal was treated with intravenous flunixin meglumine (1.1 mg/kg bwt q12 h) and cefquinome (1 mg/kg bwt q24 h) for the concurrent aspiration pneumonia. Intravenous Ringer's lactate with 2% glucose at 100 ml/hr was also initiated to correct the hypoglycemia. Blood glucose was monitored every 4–6 hours.

An esophagomyotomy was performed to correct the stricture. The foal was anesthetized with propofol at 4 mg/kg for induction and maintained with a continuous rate infusion (CRI) of propofol at 0,4 mg/kg/minute. The foal was placed in right lateral recumbency and the surgical site was prepared aseptically. An 8 cm long skin incision was made in the proximal neck area, ventral and parallel to the left jugular vein. The sternocephalic and brachiocephalic muscles were separated and the deep cervical fascia was incised to expose the esophagus. The esophagus was intubated with a nasogastric tube to facilitate identification of the stricture. As the stricture was identified, a 4 cm long longitudinal sharp incision starting 2 cm cranial from the stricture was made through the adventitia and tunica muscularis. The tunica muscularis was subsequently separated from the submucosa. The tunica muscularis and adventitia were left unsutured. Muscle layers and skin were sutured in three layers. Postoperatively the foal was only allowed to nurse and solid feed was withheld by means of a muzzle. Treatment with flunixin meglumine (1,1 mg/kg bwt q12 h) and cefquinome (1 mg/kg bwt q24 h) was continued for 5 more days. No regurgitation of milk or nasal discharge was seen after surgery. The foal was discharged from the hospital with instructions to gradually reintroduce the foal to solid food after 2 weeks.

Forty days after discharge the foal was presented to the clinic again as regurgitation of food material reoccurred. The foal was attentive, had gained 6 kilograms of bodyweight, and was in a satisfactory body condition. Physical examination revealed no abnormalities. The incision had healed without complications. Nasogastric tubing found resistance at the same location as previously and plain radiographs confirmed persistence of the stricture.

**Figure 4:**
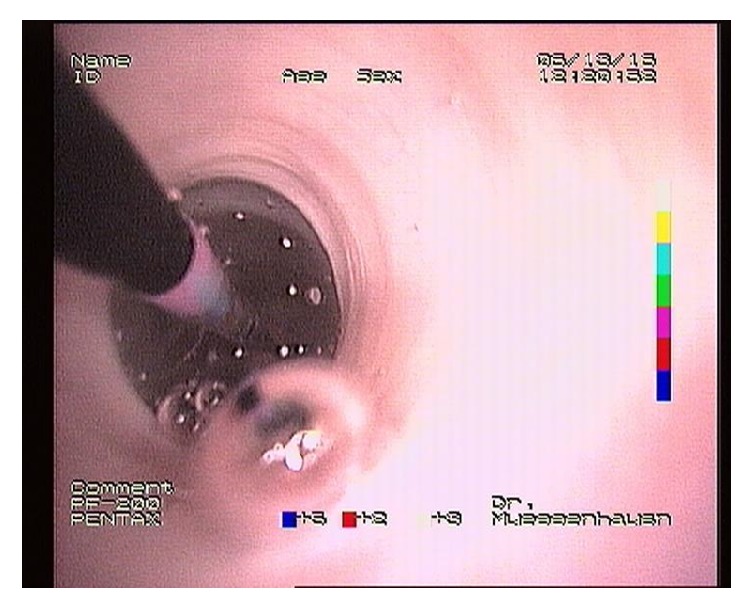
Endoscopic view of the esophagus. The balloon catheter can be seen in place, fully dilated.

**Figure 5:**
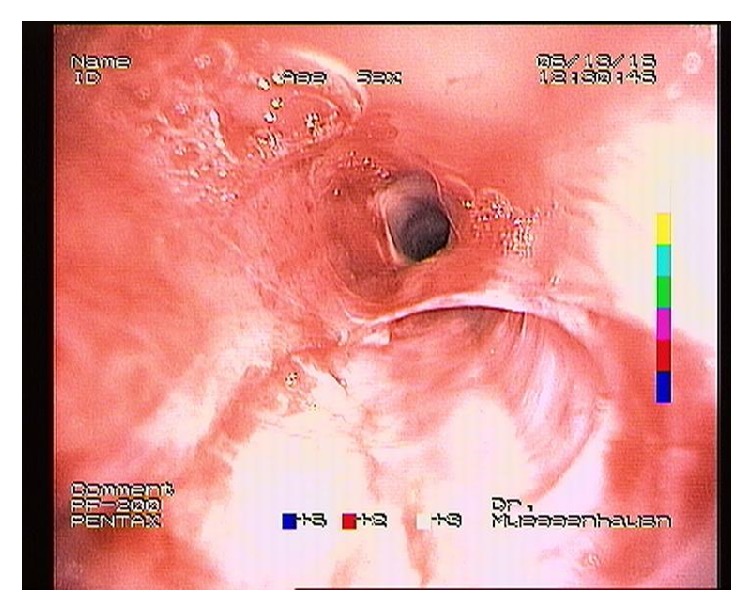
Endoscopic view of the esophagus immediately after balloon dilation. Slight hemorrhage can be observed.

As the esophageal stricture had not improved sufficiently, treatment by balloon dilation was initiated. After induction with 4 mg/kg propofol general anesthesia was maintained with a propofol CRI at 0,4 mg/kg/min. An endoscope was passed into the esophagus to evaluate the interior and identify the location of the stricture. Apart from the stricture no abnormalities were visible. Subsequently a 240 cm long balloon catheter (CRE™ PRO Wireguided, Boston Scientific) was placed into position by advancing it through the working canal of the endoscope (Figures 4 and 5). During three separate sessions with 10-day intervals the stricture was dilated twice for 30 seconds after which the balloon catheter was removed. Over the three sessions the pressure and thus diameter of the balloon were gradually increased from 10 up to 20 mm. The diameter of the balloon was monitored through an inflation device (Encore™ 26 Inflation Device, Boston Scientific) attached to the balloon catheter. After deflation the stricture site was evaluated endoscopically and topically treated with 3 ml 2% dexamethasone to reduce possible swelling and scarring of the esophageal wall (Figure 5). The foal received flunixin meglumine intravenously once at 1.1 mg/kg. One day after the procedures the foal was discharged with instructions to restrict solid feed intake.

Two days after the second balloon dilation was performed the foal was presented to the clinic again as it had fever (39,7°C) and apathy and was anorexic. Physical examination showed an elevated heart rate of 100/minute and a respiratory rate of 28/minute. Nonpainful edema was observed at the left side of the neck. Treatment with intravenous flunixin meglumine (1,1 mg/kg bwt q12 h) and intramuscular procaine-penicillin (40 mg/kg bwt q24 h) was initiated. With treatment the foal improved markedly. After 2 days medication was stopped and the foal discharged.

**Figure 6:**
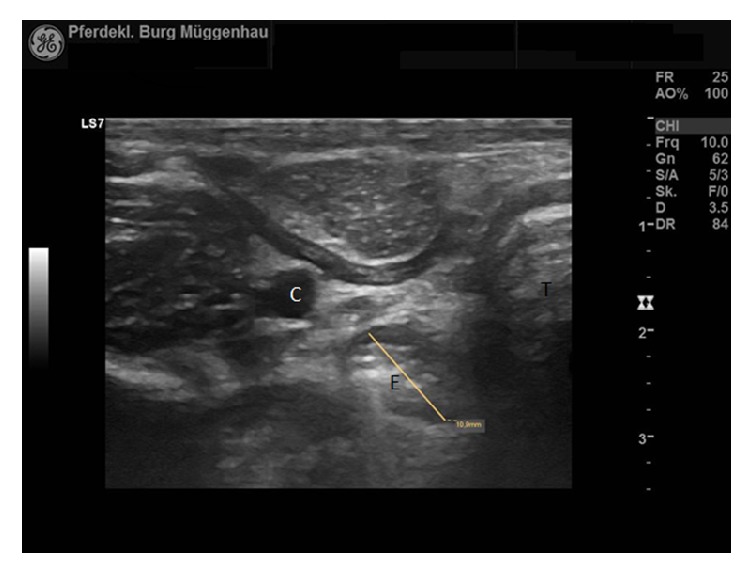
Ultrasound image of the stricture site at final follow-up examination. Diameter was measured at 10,9 mm. Left in the image is dorsal. C: carotid artery. E: esophagus. T: trachea (probe frequency 10 MHz, depth 3,5 cm).

After the third dilation the foal was increasingly allowed to eat solid food, predominantly grass and hay, over the course of two weeks. The foal did not have any complaints for up to 12 months after the final treatment. Endoscopic and ultrasonographic examination at that time showed no apparent abnormalities. The diameter at the stricture site had increased from 5 mm to 10,9 mm as assessed by ultrasonography (Figure 6).

## 3. **Discussion**

This case report describes the successful treatment of an esophageal stricture with surgery and balloon dilation in a one-month-old Miniature Shetland colt. In this particular case only the adventitia and tunica muscularis were considered abnormal; therefore a type 1 stricture was considered the most likely diagnosis. Treatment of choice for these strictures is esophagomyotomy as surgical treatment reportedly has significantly higher survival rates than nonsurgical treatment [15].

The foal did not show any signs of esophageal dysfunction up to 40 days postoperatively when regurgitation of feed reoccurred. A likely cause of the recurrence is reformation of the stricture, a common complication of esophageal surgery. Another explanation may be that the initial lesion progressed after surgery.

The foal initially presented with aspiration pneumonia as a complication to the stricture. The foal quickly improved with antimicrobial therapy and showed no further lung-associated complaints. The aspiration pneumonia was therefore not further investigated.

The most likely explanation for the fever and swelling after the second session of dilation was a transient inflammation of the dilation site with possible short term infection which quickly resolved with antibiotic and anti-inflammatory treatment.

In general, prognosis for esophageal stricture is guarded. Craig et al. [15] reported a survival rate of 22% in non-surgically managed esophageal strictures and 46% in surgically managed cases. Balloon dilation of esophageal strictures has been described in literature, mainly in case reports [10–12]. Chiavaccini and Hassel [6] describe a survival rate of 56% in patients treated with balloon dilation and that survivors could return to a normal diet afterwards.

To our knowledge this is the first report describing this abnormality in a Miniature Shetland foal. The combination of both surgical intervention and balloon dilation also has not been described previously. This case report shows that if surgical treatment for esophageal stricture is elected but shows recurrence of the problem, balloon dilation may still be performed successfully afterwards.

## Figures and Tables

**Table 1:** Normal values obtained from [14].

	Value	Reference range
WBC (×10^3^ cells/*μ*L)	13,87 × 10^9^	5,3–12,2
Glucose (mmol/L)	3,04	7,2–12

## Additional Points

*Materials*. They are as follows: CRE PRO Wireguided Balloon dilation catheter, Boston Scientific Corporation, Marlborough, MA, USA; Encore 26 Inflation Device, Boston Scientific Corporation, Marlborough, MA, USA; Micropaque Suspension, Guerbet GmbH, Sulzbach, Germany.
